# Comparative transcriptome analysis on the synthesis pathway of honey bee (*Apis mellifera*) mandibular gland secretions

**DOI:** 10.1038/s41598-017-04879-z

**Published:** 2017-07-03

**Authors:** YuQi Wu, HuoQing Zheng, Miguel Corona, Christian Pirk, Fei Meng, YuFei Zheng, FuLiang Hu

**Affiliations:** 1 College of Animal Science, Zhejiang University, Hangzhou, 310058 P.R. China; 2 USDA-ARS Bee Research Laboratory, Beltsville, MD USA; 3 Social Insect research Group, Department of Zoology and Entomology, University of Pretoria, 0002 Pretoria, South Africa

## Abstract

Secretions from mandibular glands (MGs) have important caste-specific functions that are associated with the social evolution of honey bees. To gain insights into the molecular architecture underlying these caste differences, we compared the gene expression patterns of MGs from queens, queenright workers (WQRs) and queenless workers (WQLs) using high-throughput RNA-sequencing technology. In total, we identified 46 candidate genes associated with caste-specific biosynthesis of fatty acid pheromones in the MG, including members of cytochrome P450 (CYP450) family and genes involved in fatty acid β-oxidation and ω-oxidation. For further identification of the CYP450s genes involved in the biosynthesis of MG secretions, we analyzed by means of qPCR, the expression levels of six of the CYP450 genes most abundantly expressed in the transcriptome analysis across different castes, ages, tasks and tissues. Our analysis revealed that *CYP6AS8* and *CYP6AS11*, the most abundantly expressed CYP450 genes in worker and queen MGs, respectively, are selectively expressed in the MGs of workers and queens compared to other tissues. These results suggest that these genes might be responsible for the critical bifurcated hydroxylation process in the biosynthesis pathway. Our study contributes to the description of the molecular basis for the biosynthesis of fatty acid-derived pheromones in the MGs.

## Introduction

Reproductive division of labor in social insects is often associated with phenotypic plasticity, whereby a single genome expresses different phenotypes with marked differences in reproduction and morphology in response to environmental cues^1^. In honey bees, differential nutrition provided by nurse bees during early larval stages, determine the development of reproductive queen and sterile worker phenotypes^2^. One of the most fascinating examples of differential gene expression associated with organ plasticity in social insects is the caste-specific difference in the biosynthesis of pheromones in honey bee mandibular glands (MGs).

A major physiological differences between honey bee queen MGs and worker MGs relies on the function, which develop in both castes but serve different functions^3^: In queens, MGs are responsible for the production of queen mandibular pheromone (QMP), which mainly consists of two ω-1-hydroxylated decenoic acids (9-oxo-2-decenoic acid (9-ODA) and 9-hydroxy-2-decenoic acid (9-HDA) and two aromatic components^4^. QMP regulates critical traits in honey bee social organization based on reproductive division of labor and worker division of labor^5, 6^, including the induction of worker retinue response^7^, the attraction of drones^8^ and the inhibition of worker ovary development^9^. In contrast, worker MGs mainly produce ω-hydroxylated decenoic acids^10^, including 10-hydroxy-2-decenoic acid (10-HDA) and its precursor 10-hydroxy-decanoic acid (10-HDAA), which account for 60–80% of the total fatty acid composition of royal jelly^11, 12^ and influence larval growth^13^.

**Figure 1:**
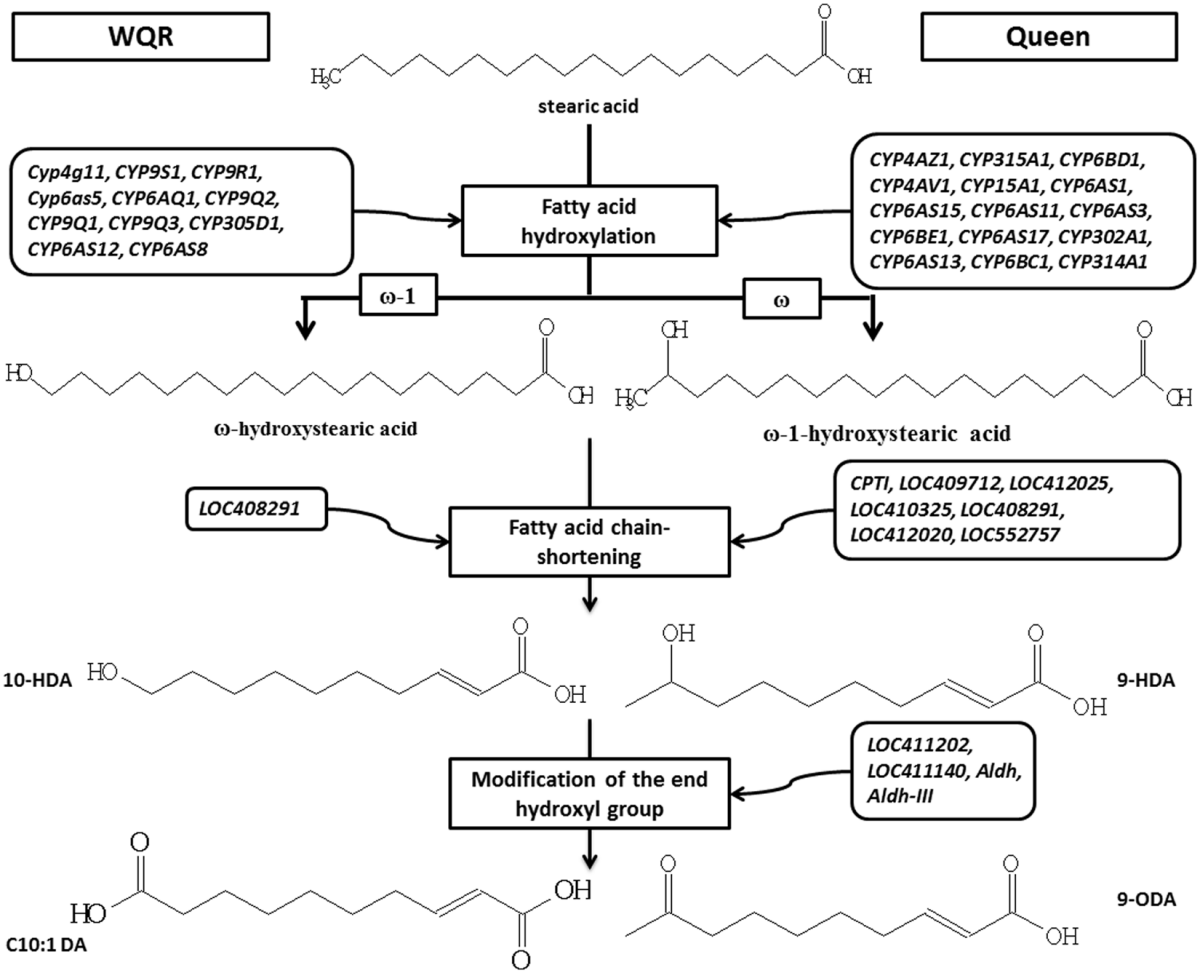
The putative biosynthesis pathway of honey bee mandibular gland secretions and possible related DEGs. Genes listed on the right side are the DEGs highly expressed in Queen library comparing to WQR library, genes on the left side are those highly expressed in WQR. Modified from Plettner *et al*.^14^.

Studies with deuterated substrates have revealed that these fatty acid-derived pheromones are synthesized through a three step bifurcated pathway^14^ as shown in Fig. 1. The synthetic precursor stearic acid is firstly hydroxylated at the 17th or 18th position, then the 18-carbon hydroxyl acids are further chain shortened via β-oxidation. The resulting 10-carbon hydroxyl acids are oxidized in a caste-selective manner, leading to the queen components (ω-1-hydroxylated) or the worker components (ω-hydroxylated)^14^. Interestingly, the production of MG fatty acid-derived pheromones is not a rigid trait but shows plasticity within the female phenotypes. Under queenless condition, some workers with active ovaries may start to produce queen-like substances^15, 16^. The MGs secretions from virgin queens contain both queen and worker components^17^.

Several studies have been conducted to elucidate the molecular basis of caste-specific MG functions. With proteomic approaches, Hasegawa *et al*. and Inovinella *et al*. have identified several differentially expressed proteins between queen MGs and worker MGs^18, 19^. Malka *et al*. presented the first global gene expression analysis of honey bee MGs from virgin queens and three types of workers using microarray analysis^20^. These studies offer the first demonstrations of genes regulating primer pheromone biosynthesis in the honey bee, and demonstrated that MG gene expression is influenced by caste, social environment and reproductive physiology. However, the important regulatory genes involved in the biosynthesis of fatty acid-derived pheromones remain to be identified.

In this study, we took advantage of high-throughput RNA-sequencing technology, which has higher coverage of all the transcribed genes and higher sensitivity compared to two-dimensional gel electrophoresis or microarrays^21^. The gene expression patterns of MGs from mated queens (Queens), queenright workers with inactivated ovaries (WQRs) and queenless workers with active ovaries (WQLs) were compared, aiming to further explore the key genes related to the biosynthesis of MG fatty acids. We found clear expression differences between our MG samples, and identified a set of differentially expressed genes (DEGs) that are potentially involved in pheromone biosynthesis and play a critical role in the regulation of complex social behaviors. Among these DEGs, we identified 34 members of the cytochrome P450 (CYTP450) family, which comprise a wide group of heme-thiolate proteins that function as oxidases in various biosynthesis processes. In insects, they are involved in many important tasks, including the synthesis and degradation of fatty acids, derived hormones and pheromones^22–24^.

In order to identify the CYP450s involved in the biosynthesis of MG secretions in honey bees, from all the CYP450s detected in the transcriptome analysis, we further analyzed the expression profile of six abundantly expressed CYP450 genes across different castes, ages, tasks and tissues.

## Results and Discussion

### Raw data processing

Approximately 6.0, 5.9 and 5.7 million clean reads, accounting for 96.42%, 98.63% and 95.2% of the raw reads were obtained from the Queen, WQR and WQL libraries, respectively. Of these total clean reads, 66.14%, 60.44% and 64.16%, were successfully matched either to a single or multiple genomic locations for each library (Table 1). Sequencing saturation analysis was used to determine the degree of sequencing depth in our study. It revealed that when the sequencing concentration of the three libraries were close to 3 M, the number of detected genes reached values close to the limit (Supplementary Figure S1), demonstrating that a sufficient sequencing depth was obtained in our analysis.

**Table 1:** Summary of mapping results based on the RNA-sequencing data from three mandibular glands libraries.

	Queen		WQL		WQR	
	reads number	percentage	reads number	percentage	reads number	Percentage
raw reads	6273609	—	5999038	—	6027842	—
clean Reads	6048878	100.00%	5711376	100.00%	5945373	100.00%
Total Mapped Reads	4000525	66.14%	3451683	60.44%	3814501	64.16%
perfect match	3018001	49.89%	2803505	49.09%	3197615	53.78%
<=2 bp mismatch	982524	16.24%	648178	11.35%	616886	10.38%
unique match	3636343	60.12%	2778087	48.64%	3224024	54.23%
multi-position match	364182	6.02%	673596	11.79%	590477	9.93%
Total Unmapped Reads	2048353	33.86%	2259693	39.56%	2130872	35.84%

WQR, queenright normal worker; WQL, ovarian active queenless worker.

We measured gene expression by counting reads that mapped to the exon regions (≥3 per gene). In total, 10309 genes were detected in at least one of the three libraries, and in each library, 9948, 9582, and 9212 expressed genes were detected respectively (Supplementary Figure S2). The most abundantly expressed genes in each library are listed in Table S1. The global gene expression patterns were practically identical in our three groups. There were 8801 genes expressed in all three groups, while only a small portion of genes were expressed in two groups or exclusively in a single group (Supplementary Figure S2). This suggests that the differences in MG’s functions are mainly regulated by differential gene expression rather than the regulation of specific genes.

**Figure 2:**
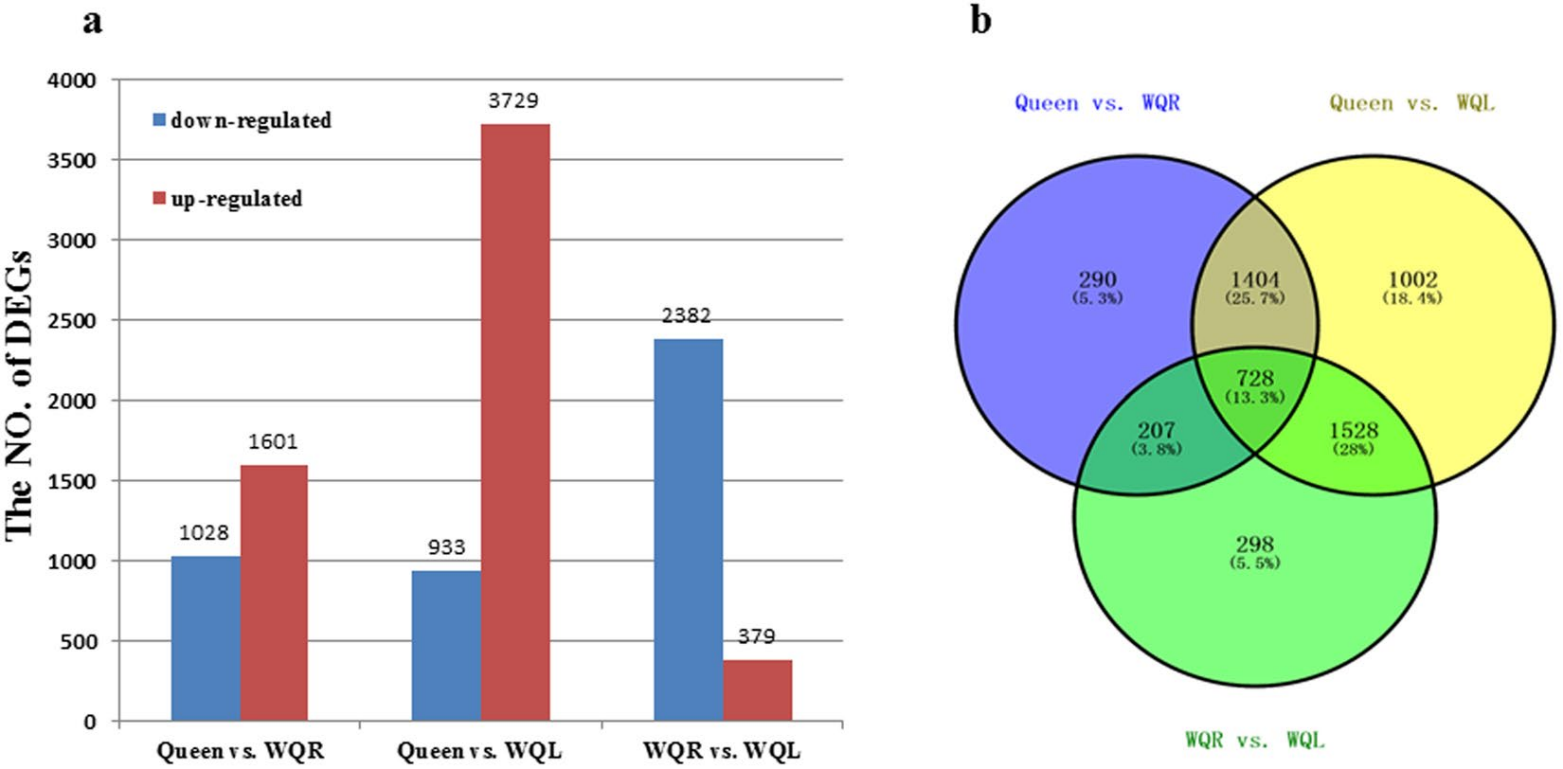
The DEGs among the three libraries. (**a**) Histogram of the DEGs from the three libraries. In each comparison (**a** vs. **b**), upregulated means that these genes were highly expressed in the latter group, (**b**) while downregulated means that genes were highly expressed in the former (**a**). (**b**) Venn diagram of the DEGs among the three libraries.

A total of 5457 transcripts were differentially expressed in the Queen vs. WQR, WQR vs. WQL and Queen vs. WQL comparisons (Fig. 2). In the Queen vs. WQR comparison, 1601 genes were highly expressed in queen MGs and 1028 genes were highly expressed in WQR MGs. After queen loss, 2382 genes were downregulated while 379 genes were upregulated in the WQL MGs. Interestingly, the greatest difference on the expression profile was found between Queen and WQL, with 3729 genes highly expressed in queen MGs and 933 highly expressed in WQL MGs (Fig. 2). Of all these transcripts, we have identified 46 DEGs potentially involved in the biosynthesis and transportation of fatty acids.

### Fatty acid hydroxylation

**Figure 3:**
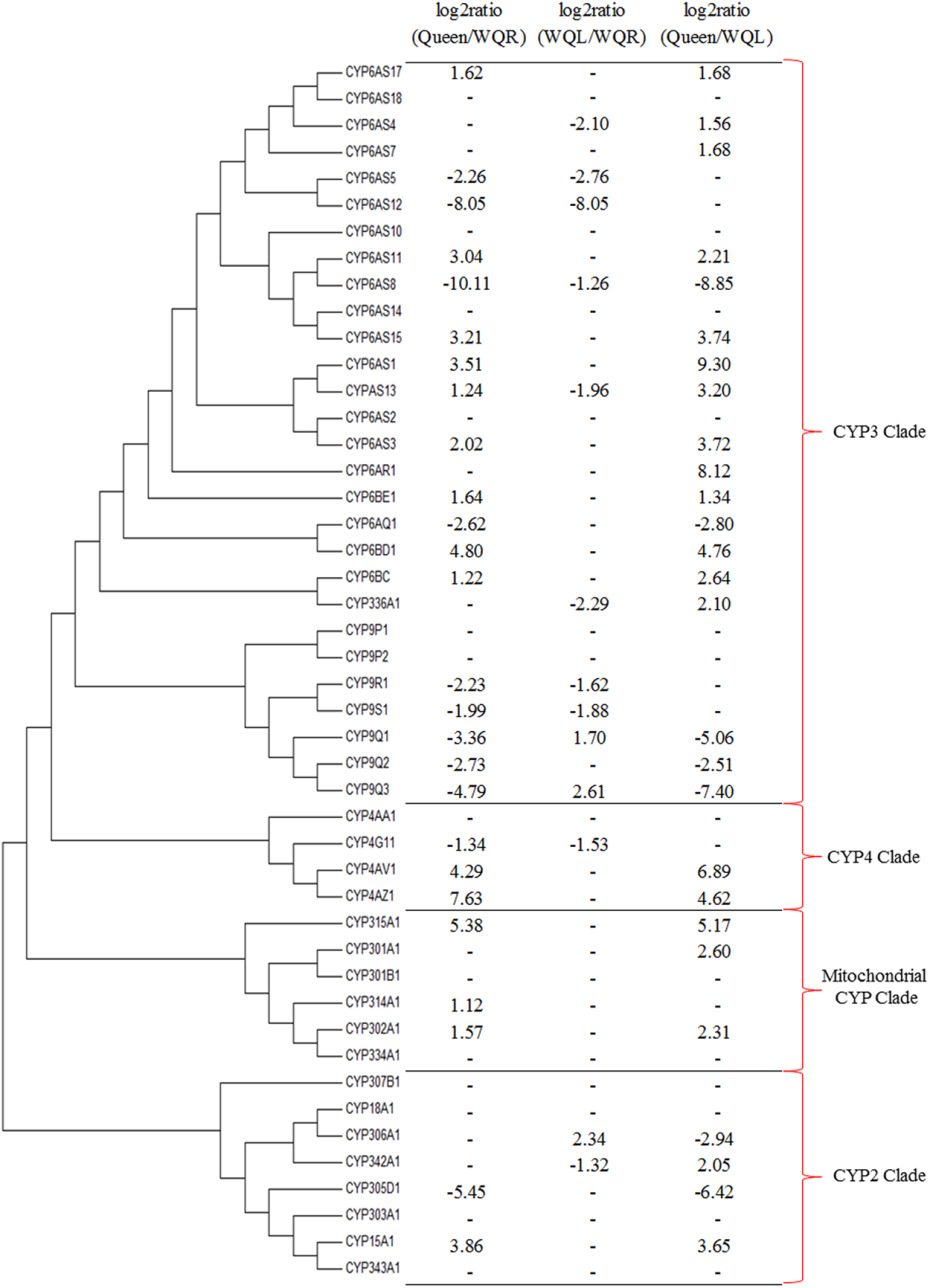
Neighbor-joining phylogeny for the honey bee P450 sequences and the expression pattern of P450 genes. RPKM was used to calculate the log2ratio (A/B). The positive number means that this gene was highly expressed in the latter group (B) and the negative number means this gene was highly expressed in the former group (A). “–” means that the log2ratio does not fit the statistical criteria (|log2Ratio| ≥ 1 and FDR ≤ 0.001) and that the gene was not differentially expressed.

The hydroxylation of stearic acid is considered to be catalyzed by the CYP450^25^, Of the 46 putatively functional CYP450 genes encoded in the honey bee genome^22^, 34 genes were differentially expressed (Fig. 3), among them, 26 were differentially expressed between the Queen and WQR, with 15 highly expressed in the Queen and 11 in the WQR library. After queen loss, of the 13 differentially expressed CYP450s between WQR and WQL, 3 were upregulated and 10 were downregulated.

In *Drosophila melanogaster* ^24^, CYP6A8, a member of the CYP6 family, was found to hydroxylate the ω-1 position of lauric acid, demonstrating that members of the CYP6 family participate in fatty acid hydroxylation in insects. Our results reveled that *CYP6AS11* was the most abundantly expressed CYP450 gene in the MGs queen library, with higher levels relative to WQR MGs. In contrast, *CYP6AS8* was highly expressed in WQR MGs, but its expression was barely detected in queen MGs (Supplementary Table S1). A similar result was found between the virgin queens and workers^20^. After queen loss, *CYP6AS8* was significantly downregulated in WQL compared to WQR, which coincides with its functional shift. The large expression differences between castes, together with the changes in expression after queen loss found in the transcript libraries, suggests that these two members of the CYP6 family play an important role in the biosynthesis of MG secretions and are possibly involved the hydroxylation of fatty acid pheromone precursors in honey bee MGs.

Previously others CYP450 genes have been implicated in the hydroxylation of fatty acids in honey bee MGs. In a qPCR study, *CYP4AA1* and *CYP18A1* were found differentially expressed in queen and worker MGs. This led to the proposal that these genes are responsible for ω-hydroxylation and ω-1 hydroxylation, respectively^26^. However, in a subsequent microarray study, no further support for this proposal was obtained due to the low expression levels of these genes^20^. Similarly, in our data, these genes were not differentially expressed in any of the comparisons and their expression levels were low in all three groups.

### Fatty acid chain-shortening

After hydroxylation, the fatty acid carbon chain is shortened through the β-oxidation process, leading to the production of decanoic and decenoic acids. This limited chain shortening is a key step in pheromone in honey bees and other insect species^25, 27, 28^, and is considered a putative regulatory point^25^.

β-oxidation happens in two organelles - mitochondria and peroxisome. One β-oxidation cycle contains four reactions, including acyl-CoA β-dehydrogenation, 2-trans-enoyl-CoA hydration, further dehydrogenated to 3-ketoacyl-CoA, and 3-ketoacyl-CoA thiolysis, and the whole process is controlled by rate-limiting enzyme.

In total, 5 genes related to mitochondrial β-oxidation and 3 genes belong to peroxisomal β-oxidation were differentially expressed (Table 2), and the rate-limiting enzymes in this two systems, carnitine O-palmitoyltransferase (CPT) and peroxisomal acyl-coenzyme A oxidase (ACOX), were found to be highly expressed in queen MGs.

**Table 2:** Comparison of the differentially expressed genes involved in fatty acid degradation.

	GeneID	Blast nr	log2ratio (Queen/WQR)	log2ratio (WQL/WQR)	log2ratio (Queen/WQL)
FA β-oxidation (mitochondrial)	*CPTI*	carnitine O-palmitoyltransferase 1, liver isoform	1.12	−1.10	2.22
	*LOC409712*	short/branched chain specific acyl-CoA dehydrogenase, mitochondrial-like	2.59	—	2.55
	*LOC412025*	very long-chain specific acyl-CoA dehydrogenase, mitochondrial-like	1.59	—	2.22
	*LOC410325*	trifunctional enzyme subunit alpha, mitochondrial-like	1.18	—	2.40
	*LOC408291*	3-ketoacyl-CoA thiolase, mitochondrial-like	−1.26	—	—
FA β-oxidation (peroxisomal)	*LOC412020*	peroxisomal acyl-coenzyme A oxidase 3-like	2.96	2.01	—
	*LOC552757*	probable peroxisomal acyl-coenzyme A oxidase 1	1.75	—	3.03
	*LOC409986*	peroxisomal multifunctional enzyme type 2-like	—	−1.14	1.55
FA ω-oxidation	*LOC411202*	alcohol dehydrogenase [NADP+] A-like	4.46	—	5.57
	*LOC411140*	putative aldehyde dehydrogenase family 7 member A1 homolog isoform 2	3.60	—	4.45
	*Aldh*	aldehyde dehydrogenase, mitochondrial isoform 1	1.94	−2.26	4.20
	*Aldh-III*	aldehyde dehydrogenase family 3 member B1	1.20	—	1.21

Blast nr, results of blast against nr database; log2ratio (A/B), RPKM was used to calculate the log2ratio (A/B), positive number means this gene was highly expressed in latter group (B) and negative number means this gene was highly expressed in former group (A), “−” means log2ratio does not fit statistical criteria (|log2Ratio| ≥ 1 and FDR ≤ 0.001) and this gene was not differentially expressed.

In the mitochondrial system, the first step in this process is the uptake of acyl-CoA by CPT, which controls the movement of fatty acid from the cytosol into the intermembrane space of mitochondria^29^. On the other hand, in peroxisomal β-oxidation, ACOXs catalyze the initial step and is considered the main enzymatic step that controls the flux through the pathway^30^.

Queen produces considerably higher quantities of fatty acid-derived pheromones in the MGs compared to workers^17^. The high expression of these rate-limiting enzymes observed in queen MGs, indicates that the higher biosynthetic capability of queen compared to worker MGs is due to the higher rate of β-oxidation.

### Modification of the end hydroxyl group

The modification of the end hydroxyl group by ω-oxidation is the final step in the secretion biosynthesis process and is crucial to the production of several important end products, such as 9-ODA from 9-HDA and sebacic acid from 10-HDAA. All four DEGs associated with ω-oxidation were highly expressed in queens (Table 2), including a key gene *LOC411202*, that encodes alcohol dehydrogenase. This enzyme catalyzes the oxidation of hydroxyl groups, and could have a critical role in the conversion of 9-HDA to 9-ODA.

The high expression of this gene in queen MGs is consistent with the caste-specific characteristic that the queen MGs mainly produce 9-ODA, while worker MGs only produce a small amount of ω-oxidized fatty acids^17^. This gene was also found to be highly expressed in virgin queen MGs compared to workers^20^ and was selectively expressed in queen MGs at the protein level^18^. These results reveal the possible molecular mechanism why queens produce a high ratio of ω-oxidized fatty acids, while workers produce hydroxyl acids.

### Transportation of fatty acids and lipids

Transportation of fatty acids and lipids is a crucial process for their uptake and utilization, as well as for the secretion of MG final products. Two DEGs that code for fatty acid binding protein, two for fatty acid transport protein and three for apolipophorin were identified among the groups (Supplementary Table S2). It is noteworthy that the apolipophorin-III coding gene *A4*, was abundantly expressed in honey bee MGs, as the fifth most abundant gene among all the detected genes (Supplementary Table S1) in each of the three libraries. Moreover, its expression was significantly higher in WQR than in Queen, and no difference was found between WQL and WQR. Apolipophorin-III is an important lipid transport protein in insects^31^. It serves to stabilize the DAG-enriched particles by providing an interface between surface-localized hydrophobic DAG molecules and the external aqueous medium. Physical studies on *M. sexta* apolipophorin-III have shown that it has a high affinity for both phospholipid and diacylglycerol surfaces^32^, which are the major lipid components in haemolymph transported in insects^33^. The high expression of this protein may be relevant for the secretion of fatty acid components or the transport of phospholipids and glycerides as fatty acid resources for biosynthesis.

Small chemosensory proteins (CSPs) and odorant binding proteins (OBPs) are two major classes of soluble proteins involved in chemical communication^34–36^. CSPs and OBPs are expressed not only in insect sensory organs, but also in other tissues that lack gustatory and olfactory neurons^36, 37^. It has been hypothesized that some OBPs and CSPs participate in the MG secretion biosynthesis pathways^19, 20^. Of the expressed OBP and CSP genes, *CSP3* showed elevated expression levels (Supplementary Table S1) in all three libraries. A ligand binding experiment showed that CSP3 binds specifically to large fatty acids and ester derivatives^38^ similar to the biosynthesis precursor - stearic acid, suggesting that CSP3 might work as a fatty acid transporter in honey bee MGs.

### qPCR expression analysis of CYP450 genes

Due to the high diversity of CYP450s in sequence, function and substrates, it is complicated to characterize their specific functions. However, profiling the expression pattern of CYP450s in different organs and developmental stages can shed light on their specific functions^39, 40^.

**Figure 4:**
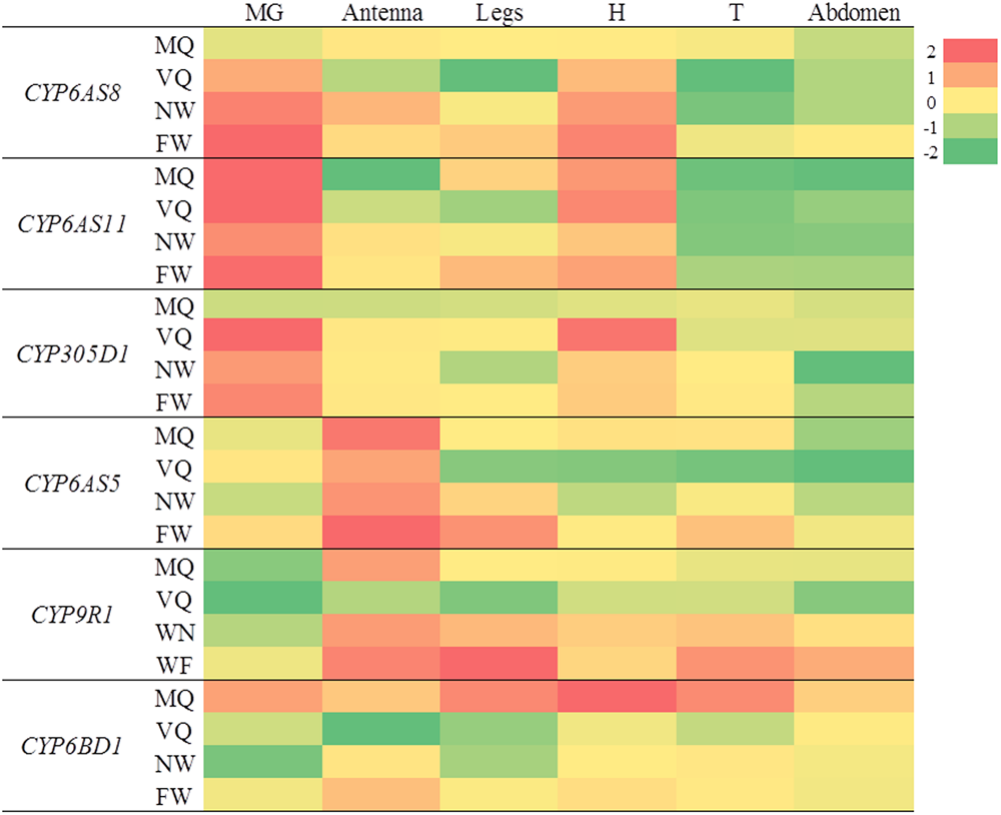
Heat map representing the expression patterns of CYT*P450* genes. MGs from MQ was used as reference sample and the relative expression levels were log2 transformed. Experimental groups: Mated queen (MQ), virgin queen (VQ), nurse worker (NW) and forage workers (FW). Organs and segments: Mandibular gland (MG), antenna, leg, head without mandibular gland and antenna (H), thorax without legs (T) and abdomen were collected and analyzed using quantitative PCR (qPCR).

Based on the transcriptome analysis, we further analyzed the expression pattern of six CYP450s, to provide additional information about their functions. *CYP6AS5*, *CYP6AS8*, *CYP6AS11*, *CYP6BD1*, *CYP305D1* and *CYP9R1* were the most abundantly expressed P450s with RPKM (Reads Per Kilobase of transcript per Million mapped reads) value >100 in at least one MG group, and were all differentially expressed between queen and WQR. From mated queen (MQ), virgin queen (VQ), nurse worker (NW) and forage workers (FW), the mandibular gland (MG), antenna, leg, head without mandibular gland and antenna (H), thorax without legs (T) and abdomen were collected and analyzed using quantitative PCR (qPCR). A heat map representing the expression profile of these CY*P450* genes is shown in Fig. 4.

**Figure 5:**
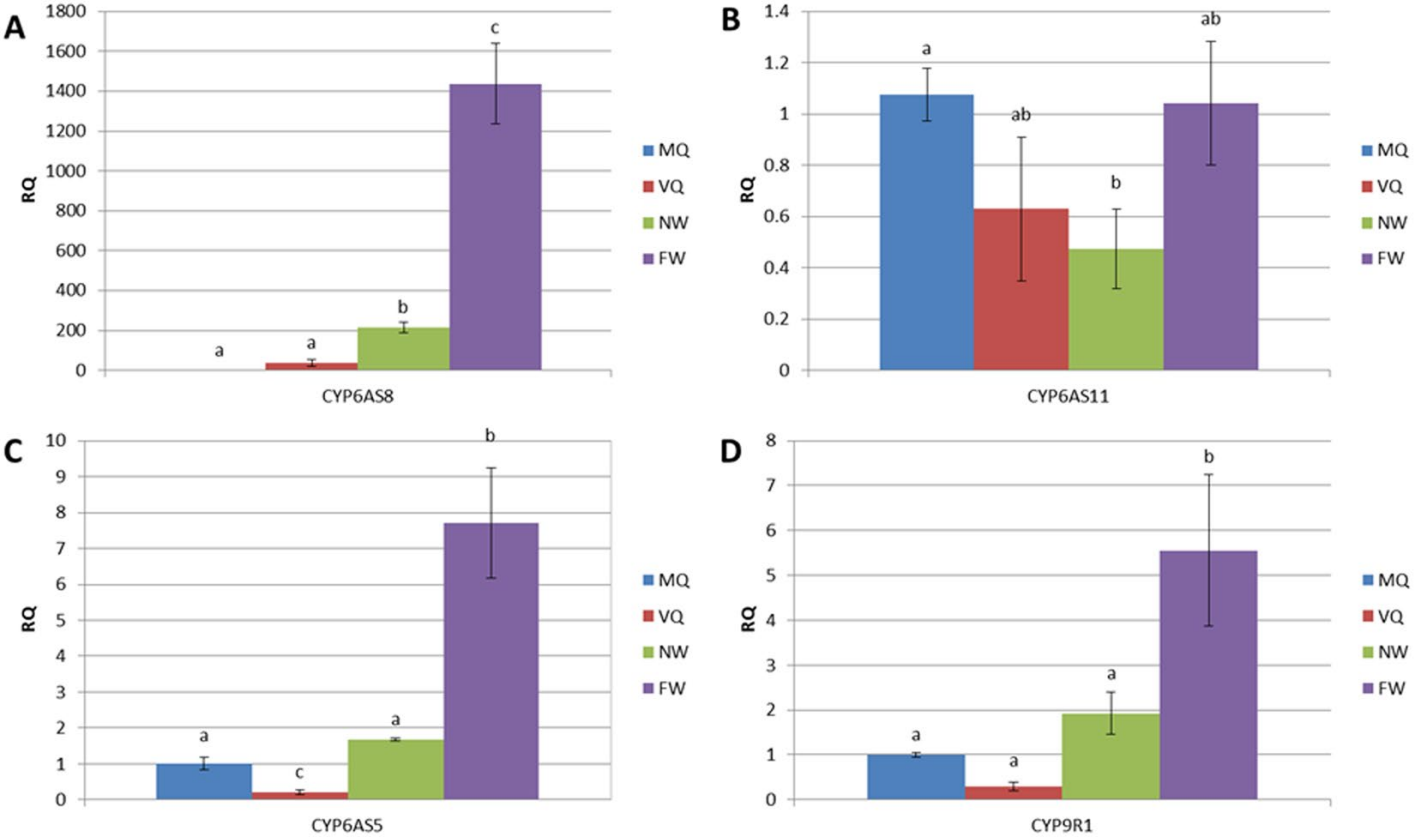
Expression levels of CYT*P450* genes among castes and physiological groups. (**A**) the expression of *CYP6AS8* in MGs. (**B**) the expression of *CYP6AS11* in MGs. (**C**) the expression of *CYP6AS5* in legs. (**D**) the expression of *CYP9R1* in legs. RQ, fold change in gene expression. Error bars represent SD fold changes. Different letters represent significant difference between groups (*p* < 0.05).

The expression of *CYP6AS8* and *CYP6AS11* were both highly enriched in MGs with a low level of expression in the head. Specifically, *CYP6AS11* was mainly enriched in the MGs and heads of all the four types of bee and barely detectable in other segments. *CYP6AS8* was highly expressed in the MGs of VQ and workers while its expression was low in all segments of MQ. The expression level of *CYP6AS8* in MGs of workers was over 100-fold higher compared to the MGs of MQ (Fig. 5a). On the other hand, the expression of *CYP6AS11* was significantly higher in the MGs of MQ compared to the MGs of NW (Fig. 5b), which is in agreement with the transcriptome data. Through qPCR analysis, the observed selective expression of *CYP6AS8* and *CYP6AS11* in MGs, along with their differences in expression between castes, further supports the idea that these genes have important functions in the biosynthesis of MG secretions.

*CYP6AS5* and *CYP9R1* were both mainly expressed in the sensory structures: antennas and legs. The high level expression of these two *CYP450*s in antennas and their upregulation in legs of FWs (Fig. 5c,d) suggested that they are involved in the metabolism of xenobiotics, which are consistent with reports that identified the upregulation of *CYP6AS5*, *CYP9R1* in honey bees after exposure to xenobiotics^41, 42^.

Compared to *CYP6AS5* and *CYP9R1*, *CYP305D1* and *CYP6BD1* were expressed in a different pattern. *CYP305D1* was most abundantly expressed in the MGs and Hs of VQs and was not significantly detectable in all segments of MQs. *CYP6BD1* was highly enriched in MQs, and in particular, predominantly expressed in Hs of MQs. However, the significance of the expression of these two CYP450 genes still could not be demonstrated and their precise functions remain to be determined.

## Conclusion

Our results revealed the global gene expression pattern of honey bee MGs, with approximately 10000 genes being detected. Gene expression in MGs is influenced by many factors, including caste, queen loss and individual fertility, with over five thousand DEGs identified.

The biosynthesis of fatty acid pheromones is a major function of honey bee MGs, and the molecular mechanism of this process, as well as the caste differences, are the main topics we aimed to investigate. The differential expression of the CYP450 genes between queens and workers under different social environments indicate that they have important functions in MGs, which could be related to the biosynthesis processes. Particularly interesting, is the finding of promising CYP450 genes as candidates for the hydroxylation of pheromone precursor fatty acids. Tree lines of evidence support the proposal that *CYP6AS8* and CYP6AS11 are involved in this process in honey bee MGs. First, the differences in expression levels between castes and after queen loss in workers shown by RNA sequencing analysis. Second, the selective expression in MGs revealed by qPCR. Finally, the phylogenetic analysis supporting that these genes belong to the CYP6 family involved in the hydroxylation of fatty acids in *Drosophila* ^24^. Although these pieces of evidence are thus consistent with our proposal, further functional assays such as *in vitro* expression or RNAi knockdown are required to confirm their functions.

The β- and ω-oxidation steps are considered possible regulation points in MG biosynthesis^25^, The DEGs we identified have revealed the molecular basis for the higher biosynthetic capability and increased oxidation ratio of secreted end products in queen compared with workers.

Laying workers are atypical individuals in a colony with a complex physiology regulated by several factors, some of them not fully characterized. MGs function in workers is affected by environmental cues (e.g., queen loss) that influence ovarian development. MGs in laying workers have a tendency to produce queen-like secretions, but this trait is likely affected by the interplay of environmental (e.g., queen loss) and genotypic components that determine ovarian development. In this study, contrary to our expectation, we found higher differences in gene expression between queen and WQL, compared to queen and WQR. Interestingly, workers of African *A. mellifera* subspecies, such as *A. mellifera scutellata* and A*. mellifera capensis*, exhibit considerably higher secretion of queen-like pheromones after queen loss compared to European subspecies^43, 44^. We hypothesize that European-derived *A. mellifera* species could have experienced a restraint in the development of queen-like traits after queen loss associated with domestication. Alternatively, our results could reveal unknown characteristics of laying worker physiology.

Our findings provide important insights into the caste-specific differences in MG function and open new avenues for scientific research on one of the more fascinating examples of organ plasticity in social insects.

## Methods

### Honey bee rearing and collection

Honey bee (*Apis mellifera ligustica*) colonies were reared at the Honey Bee Research Laboratory in the College of Animal Sciences, Zhejiang University, Hangzhou, China. To obtain workers of specific ages, we removed sealed brood combs containing emerging adult workers from four unrelated source colonies and placed them in an incubator overnight following standard protocol^45^. The following day, newly emerged workers were marked and transferred to the prepared queenright and queenless host colonies.

For comparative transcriptome analysis, honey bee mature queen samples were freely mated 1-year-old sisters. WQLs and WQRs were collected from corresponding colonies on day 14 after emerging. The abdomens of the workers were dissected to verify the reproductive status of their ovaries. Workers with activated ovaries from queenless colonies and workers with inactivated ovaries from queenright colonies were sampled following the classification of Pirk *et al*.^45^.

For expression profile analysis, honey bee MQs were freely mated 1-year-old sisters, VQs were also sisters and were collected immediately after emerging from queen cells (within 3 hours), NWs were sampled on day 6 after emerging and FWs were sampled on day 20 from queenright colonies.

### Sample preparation and data acquisition for RNA-sequencing analysis

Queen and worker honey bees were frozen at −20 °C, and their MGs were immediately dissected under a stereo microscope (SZ-61, Olympus Life Science) in RNase-free water, and placed in chilled vials. For the Queen library, 60 MGs were dissected from 30 queens and pooled together. For each worker library, 180 MGs dissected from 90 workers were pooled.

Total RNA was extracted from frozen MGs using TRIzol (Invitrogen, Carlsbad, CA, USA) according to the manufacturer’s protocol. TruSeq RNA Sample Preparation Kit (Illumina, San Diego, CA, USA) was used for cDNA library construction, the with the mRNAs being enriched from the total RNA using oligo (dT) magnetic beads and fragmented into short fragments. These short fragments were used as templates for cDNA synthesis. The cDNA libraries were then sequenced on an Illumina HiSeq^TM^ 2000. The raw data from each library was deposited in the Sequence Read Archive (SRA) of the National Center for Biotechnology Information (NCBI) with accession number SRA420958.

The original image data produced from sequencing was transferred into sequence data by base calling. Clean reads were obtained via the strategies described by Niu *et al*.^46^. The clean reads from 3 libraries were mapped to the honey bee genome (Amel_4.0)^47^ using SOAPaligner/SOAP2^48^. No more than 2-base mismatches were allowed in the alignment.

### Data analysis

The gene expression level was calculated by using the RPKM method^49^. All libraries of clean reads were normalized to RPKM value. A strict rigorous algorithm was performed to identify DEGs between two samples, the threshold with a FDR (False Discovery Rate) ≤ 0.001 and the absolute value of log2Ratio ≥ 1 was used to judge the significance of gene expression differences^50^.

We performed a cluster analysis on the gene expression patterns with cluster^51^ and Java Treeview software^52^ shown in Supplementary Figure S3. Venn diagrams were constructed using Venny software^53^. Phylogenetic analysis of the P450 proteins from *A. mellifera* were conducted using the MEGA version 6 program^54^ with the alignment generated by the DNAMAN version 6 program and a neighbor-joining tree created with 1000 bootstrap replications.

### Sample preparation and RNA extraction for the expression analysis of P450s

For gene expression comparison of the P450s of interest, 15 MQs, 15 VQs, 30 NWs and 30 FWs were collected and the experiment was replicated in three times. Honey bees were then frozen at −20 °C and dissected. Total RNA was extracted using RNApure Total RNA Kit (Aidlab Biotechnologies Co. Ltd., Beijing, China) according to the manufacturer’s protocol. The RT-PCR reaction was performed using 0.5 μg total RNA with ReverTra Ace qPCR RT Kit (Toyobo life science, Shanghai, China).

### qPCR analysis

We assayed the transcript levels of the following genes: *CYP6AS5*, *CYP6AS8*, *CYP6AS11*, *CYP6BD1, CYP9R1* and *CYP305D1*, with housekeeping gene *actin* chosen as the reference control. Intron-spanning primers were designed using Primer Premier 6.0 (Supplementary Table S3). All assays were performed in triplicates in a final volume of 10 μL. Reaction mixtures were setup with 1 μl cDNA (10X diluted), 0.5 μl of forward and reverse primers (10 μM), 5 μl Thunderbird SYBR Green qPCR Mix (Toyobo life science, Shanghai, China) and 3 μl distilled water. Transcription levels were quantified using the StepOne Plus real time PCR system. The relative expression levels of the selected genes were calculated using the 2^−∆∆Ct^ method^55^. A heatmap was constructed using Microsoft Excel 2010.

The expression levels of the DEGs obtained from RNA-sequencing were also validated using qPCR analysis. cDNA samples from RNA-sequencing analysis were used, and the relative expression level of following genes: *PLA2-2.2, PLD, LOC412020, CYPA4Z1, CYP4AA1, Vg* were quantified as described above. The qPCR analysis results were consistent with the data obtained from the RNA-sequencing (Supplementary Table S4).

### Statistical analysis

Statistical analysis was carried out using SPSS software version 19.0. For the validation of RNA-sequencing, the correlation coefficient was used to evaluate the correlation of qPCR data and RNA-sequencing data. For the expression profiling, statistical significance was calculated using one-way analysis of variance following LSD method or Dunnett’s T3 method, *P* values < 0.05 were considered as statically significant.

## Electronic supplementary material

Supplementary information
