# Supplementary material for: Comparative transcriptome analysis on the synthesis pathway of honey bee (Apis mellifera) mandibular gland secretions
Source: Sci Rep. 2017 Jul 3;7:4530. doi: 10.1038/s41598-017-04879-z (PMC5495765; doi:10.1038/s41598-017-04879-z)
Supplement: Supplementary file 1 — Supplementary information [file 41598_2017_4879_MOESM1_ESM.doc]

Comparative transcriptome analysis on the synthesis pathway of honey bee (*Apis Mellifera*) mandibular gland secretions

YuQi Wu^1^, HuoQing Zheng^1^, Miguel Corona^2^, Christian Pirk ^3^, Fei Meng^1^, YuFei Zheng^1^, FuLiang Hu^1^*

| LIBRARY | Gene Symbol | RPKM | Blast nr |
| --- | --- | --- | --- |
| Queen | *Vg* | 45292 | vitellogenin precursor |
|  | *LOC503505* | 8418.24 | icarapin-like precursor |
|  | *LOC406105* | 7687.26 | esterase A2 |
|  | *Hsc70-4* | 3780.23 | heat shock protein cognate 4 |
|  | *A4* | 3022.62 | apolipophorin-III-like protein precursor |
|  | *Rfabg* | 2725.47 | PREDICTED: apolipophorins isoform 1 |
|  | *LOC100578039* | 2465.47 | - |
|  | *LOC409708* | 2374.9 | PREDICTED: glucosylceramidase-like isoform 1 |
|  | *LOC726625* | 2149.03 | PREDICTED: luciferin 4-monooxygenase-like |
|  | *LOC100578199* | 2129.81 | RecName: Full=Icarapin-like; AltName: Full=Venom carbohydrate-rich protein; Flags: Precursor |
|  | *LOC100577586* | 2089.22 | PREDICTED: hypothetical protein LOC100577586 |
|  | *LOC100577725* | 2082.5 | PREDICTED: hypothetical protein LOC100577725 |
|  | *CSP3* | 1910.92 | chemosensory protein 3 precursor |
|  | *LOC408395* | 1860.42 | PREDICTED: venom carboxylesterase-6-like |
|  | *LOC408807* | 1707.45 | IRP30 |
|  | *Aldh* | 1645.89 | PREDICTED: aldehyde dehydrogenase, mitochondrial isoform 1 |
|  | *Amci* | 1378.2 | PREDICTED: chymotrypsin inhibitor |
|  | *CYP6AS11* | 1360.58 | PREDICTED: probable cytochrome P450 6a13 |
|  | *LOC410857* | 1338.64 | PREDICTED: protein lethal(2)essential for life-like isoform 1 |
|  | *LOC552018* | 1320.75 | PREDICTED: aldo-keto reductase family 1 member B10-like |
| WQR | *LOC406093* | 33880.7 | apisimin precursor |
|  | *LOC406105* | 31103.1 | esterase A2 |
|  | *LOC503505* | 25935.1 | icarapin-like precursor |
|  | *Mrjp1* | 18346.7 | major royal jelly protein 1 precursor |
|  | *A4* | 9252.63 | apolipophorin-III-like protein precursor |
|  | *LOC100578199* | 7548.44 | - |
|  | *CYP6AS8* | 7084.44 | PREDICTED: probable cytochrome P450 6a14 |
|  | *Ant* | 5564.39 | ADP/ATP translocase |
|  | *Mp20* | 5014 | PREDICTED: muscle-specific protein 20 |
|  | *CSP3* | 4936.62 | chemosensory protein 3 precursor |
|  | *TpnCIIIa* | 4924.25 | troponin C type IIIa |
|  | *Mlc2* | 4634.74 | PREDICTED: myosin regulatory light chain 2 |
|  | *LOC409708* | 4248.72 | PREDICTED: glucosylceramidase-like isoform 1 |
|  | *LOC410058* | 3984.14 | PREDICTED: myosin light chain alkali-like isoform 4 |
|  | *GMCOX14* | 3982.39 | PREDICTED: glucose dehydrogenase [acceptor] isoform 3 |
|  | *SP28* | 3974.92 | PREDICTED: venom serine protease 34 |
|  | *LOC408308* | 3680.28 | PREDICTED: coiled-coil-helix-coiled-coil-helix domain-containing protein 2, mitochondrial-like |
|  | *LOC411285* | 3403.66 | PREDICTED: muscle LIM protein Mlp84B-like isoform 1 |
|  | *LOC725381* | 3236.28 | PREDICTED: hypothetical protein LOC725381 |
|  | *Apd-2* | 3085.57 | - |
| WQL | *LOC406093* | 40706.3 | apisimin precursor |
|  | *Apd-2* | 23538.2 | apidermin 2 |
|  | *LOC406105* | 21254.8 | esterase A2 |
|  | *Mp20* | 18337.1 | PREDICTED: muscle-specific protein 20 |
|  | *A4* | 11480.5 | apolipophorin-III-like protein precursor |
|  | *SP28* | 11287.4 | PREDICTED: venom serine protease 34 |
|  | *LOC408308* | 10992.1 | PREDICTED: coiled-coil-helix-coiled-coil-helix domain-containing protein 2, mitochondrial-like |
|  | *LOC503505* | 10558.7 | icarapin-like precursor |
|  | *LOC406081* | 9193.37 | glucose oxidase |
|  | *LOC411285* | 9071.91 | PREDICTED: muscle LIM protein Mlp84B-like isoform 1 |
|  | *RpLP1* | 7122.15 | 60S acidic ribosomal protein P1 |
|  | *LOC725074* | 6891.23 | PREDICTED: hypothetical protein LOC725074 |
|  | *LOC725381* | 6524.5 | PREDICTED: hypothetical protein LOC725381 |
|  | *RpLP2* | 6447.93 | PREDICTED: 60S acidic ribosomal protein P2-like |
|  | *RpS2* | 6167.08 | PREDICTED: 40S ribosomal protein S2 isoform 2 |
|  | *RpL14* | 5674.2 | PREDICTED: 60S ribosomal protein L14 |
|  | *CSP3* | 5067.13 | chemosensory protein 3 precursor |
|  | *Ant* | 4935.61 | ADP/ATP translocase |
|  | *Vg* | 4674.99 | vitellogenin precursor |
|  | *Rpl41* | 4660.92 | - |
|  | *RpS17* | 4647.06 | PREDICTED: 40S ribosomal protein S17 |

**Supplementary Table S1. The top 20 high abundantly expressed genes in each library.** Blast nr, results of blast against nr database. Genes are listed according to their RPKM value in corresponding library.

|  | GeneID | Blast nr | log2ratio | log2ratio | log2ratio |
| --- | --- | --- | --- | --- | --- |
|  |  |  | (Queen/WQR) | (WQL/WQR) | (Queen/WQL) |
| FABP | *Fabp* | FABP-like protein | -1.63 | - | -2.35 |
|  | *Fabp* | fatty acid binding protein | - | 1.22 | -1.96 |
| FATP |  | | | | |
|  | *LOC408564* | PREDICTED: long-chain fatty acid transport protein 4-like | - | -1.01 | 1.904724 |
|  | *FATP* | PREDICTED: long-chain fatty acid transport protein 4 | -1.75 | -3.22 | 1.473628 |
| lipophorin | *Rfabg* | PREDICTED: apolipophorins isoform 1 | 2.75 | - | 2.711156 |
|  | *A4* | apolipophorin-III-like protein precursor | -1.61 | - | -1.92532 |
|  | *LOC100576655* | PREDICTED: apolipophorins-like | 3.53 | -1.67 | 5.195229 |

**Supplementary Table S2. Differentially expressed fatty acid binding proteins, fatty acid transport proteins and apolipophorins.** FABP, fatty acid binding protein; FATP, fatty acid transport protein; Blast nr, results of blast against nr database; log2ratio(A/B), RPKM was used to calculate the log2ratio(A/B), positive number means this gene is highly expressed in latter sample(B) and negative number means this gene is highly expressed in former sample(A), “–” means log2ratio does not fit statistical criteria (|log2Ratio|≥1 and FDR≤0.001) and this gene is not differentially expressed.

**
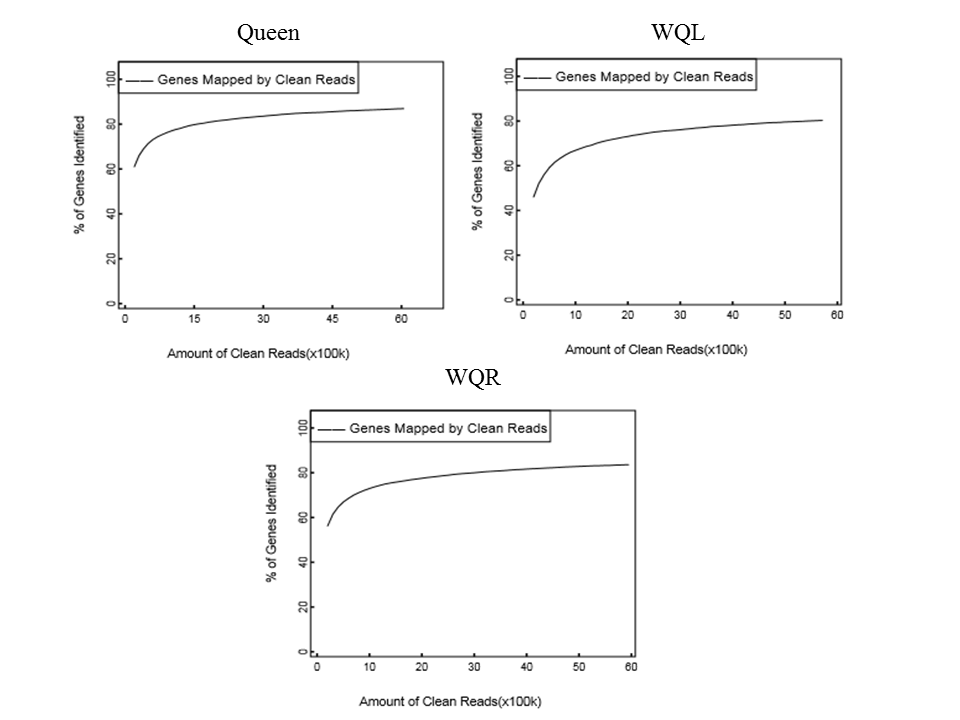
**

**Supplementary Figure. S1.** Sequencing saturation analysis of three sequenced samples.


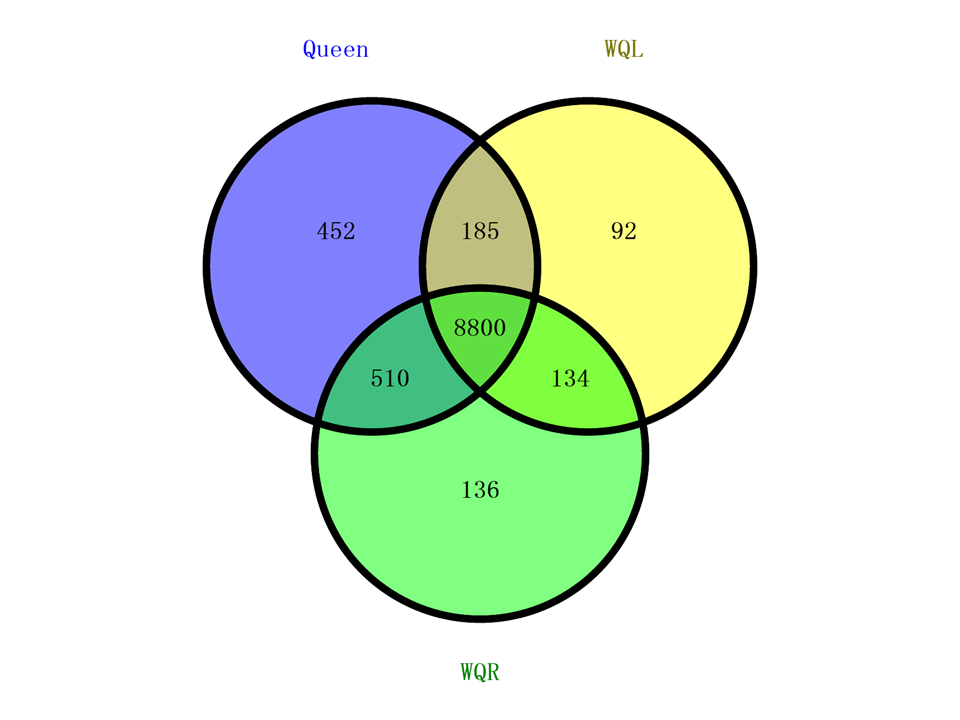


**Supplementary Figure. S2.** **The numbers of genes expressed in the MGs of queen, WQR and WQL library.** The numbers inside the diagram represent the genes either commonly or exclusively expressed among groups. The majority of genes were expressed in more than on groups, demonstrated that the differences in MG’s functions are mainly regulated by the differential expression rather than the specific gene expression.


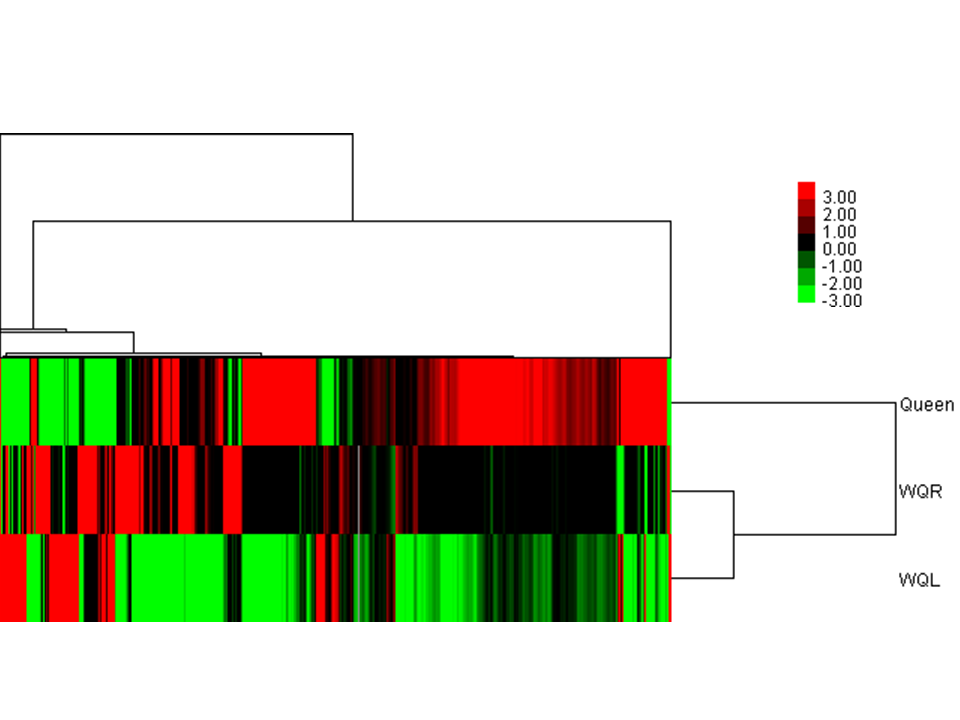
**Supplementary Figure. S3. Hierarchical clustering analysis of the DEGs from the three groups.** The red color represents upregulated genes, the green color represents downregulated genes and the grey color represents no expression in this library. Groups from the same caste were clustered into the corresponding groups, indicating that the gene expression differences between caste were greater than those between reproductive status.

|  | Gene symbol | Primer sequence |  |
| --- | --- | --- | --- |
|  |  | Forward | Reverse |
| Primers for validation | *PLA2-2.2* | AACCGATTGTGCTCCCAGTA | GTCTGGTCCGTTCTTCTTGC |
|  | *PLD* | TGGCATTCTCGACATCTTGTT | TGACTCCAAACCCATTATCCA |
|  | *LOC412020* | CCTCACTTGGCAGCAACATA | GATAACCGTGTCCTCCACAA |
|  | *CYPA4Z1* | TTGGCCGAATCCAAATAAGT | TTGTCCAATGCAATTTCGTG |
|  | *CYP4AA1* | GAGGCGTGGGTATATCTCGT | TTTCGGGCCATTTAGTTGAG |
|  | *Vg* | GGAACCTGGAACGAACAAGA | CGACGATTGGATGGTGAAAT |
| Primers for expression profile analysis |  |  |  |
|  | *CYP6AS5* | TCCGTAGAATGGGCAGAGAAGT | TCGTACAGCCTTGGCATGAATT |
|  | *CYP6AS8* | ACCGCCAATAAACTCAGAGGAATGT | ACTTCTCGCACATTGACAGGTTCTC |
|  | *CYP6AS11* | TTGCCAATTATCTCAACAGAACCAATATCC | AGAACACTCGTGAATCATGCAGAACA |
|  | *CYP6BD1* | TACTCGCACTTACTGGCTAAGAAGA | AGTGACCAGGTACTGTAGGAACAC |
|  | *CYP9R1* | GCACAGGCGTTCGTCTTCTTCT | CCCGTCCCGCTCCTCCAAA |
|  | *CYP305D1* | GGAGAATTAGTGGCGTTCAATCAAC | CAAGGGAAAGCGGCGAGAAG |
|  | *ACTIN* | TGCCAACACTGTCCTTTCTG | AGAATTGACCCACCAATCCA |

**Supplementary Table S3. Primers for qRT-PCR**

| Gene Symbol | samples | RNA-seq | qRT-PCR | correlation coefficient |
| --- | --- | --- | --- | --- |
| *PLA2-2.2* | Queen | 0.1 | 1.337928 | 0.999676 |
|  | WQL | 28.67 | 19.42712 |  |
|  | WQR | 0.41 | 1 |  |
| *PLD* | Queen | 3.42 | 1.140764 | 0.9589 |
|  | WQL | 0.52 | 0.773782 |  |
|  | WQR | 1.58 | 1 |  |
| *CYP4AZ1* | Queen | 31.9 | 32.4467 | 0.999541 |
|  | WQL | 1.3 | 1.049717 |  |
|  | WQR | 0.16 | 1 |  |
| *CYPAA1* | Queen | 3.7 | 1.148698 | 0.998093 |
|  | WQL | 0.5 | 0.482968 |  |
|  | WQR | 2.8 | 1 |  |
| *LOC412020* | Queen | 50 | 19.29293 | 0.934875 |
|  | WQL | 25.9 | 2.989698 |  |
|  | WQR | 6.44 | 1 |  |
| *Vg* | Queen | 45292 | 83.86518 | 0.998584 |
|  | WQL | 4674 | 2.620787 |  |
|  | WQR | 1230 | 1 |  |

**Supplementary Table S4. the correlation coefficient of RNA-seq data and qRT data.** The correlation between RNA-seq data and qRT data are evaluated and demonstrate the reliability of the RNA-sequencing performed. RNA-seq data are shown in their RPKM values, qRT-PCR data are shown in relative expression level, group WQR was used as reference.
